# Skin Health Information Seeking on Short Video Platforms in Indonesia: Mixed Methods Approach

**DOI:** 10.2196/93461

**Published:** 2026-07-03

**Authors:** Fathi Qushoyyi Ahimsa, Kelvin Saputra, Michelle Elizabeth Amanda Hutasoit, Putu Wuri Handayani, Hana Fitriani

**Affiliations:** 1 Faculty of Computer Science, Universitas Indonesia, Jalan Kampus, Depok, 16424, Indonesia, 021 7863419

**Keywords:** skin, health information, information seeking, short video platform, social media, Indonesia

## Abstract

**Background:**

Many users have now switched to using short video platforms as the main channel in their search for skin health information. With high internet penetration and a large market for the skincare industry, short video platforms play an important role in the “beauty discovery” process and purchase decisions. However, the increasing consumption of skin health content is also accompanied by the risk of misinformation, uneven content quality, and the dominance of creators who are not health professionals. Therefore, it is important to determine what factors affect the use of short video platforms in the search for skin health information.

**Objective:**

By adopting the stimulus-organism-response framework, health belief model, and media richness theory, this study aims to analyze the factors that influence the use of short video platforms in the search for skin health information.

**Methods:**

This study used a mixed methods approach by distributing an online survey to 603 respondents and conducting interviews with 30 interviewees. Survey data were analyzed using the covariance-based structural equation modeling method, and qualitative data were analyzed using the thematic analysis method.

**Results:**

The results of this study found that perceived usefulness (*P*=.01), attitude (*P*=.02), perceived severity (*P*=.009), and perceived susceptibility (*P*=.02) directly affected the behavior of seeking skin health information on short video platforms. Health content expressiveness (*P*=.001) and personalized health insights (*P*<.001) directly affected perceived usefulness. These findings support the media richness theory, which shows that the expressiveness of health content and personalization can increase the perception of the usability of short video platforms. Perceived interactivity has an influence on attitude (*P*<.001), which then affects skin health information seeking on short video platforms (SHEs; *P*=.02). Upward skin comparison also had a direct influence on skin stigmatization (*P*<.001). Moreover, perceived severity (*P*=.009) and perceived susceptibility (*P*=.02) have an effect on SHEs. These findings confirm the health belief model’s theory that perception of the severity of a skin problem and the perception of a person’s likelihood of developing a skin problem can improve skin health seeking behavior. However, no effect of source credibility (*P*=.17) and skin stigmatization (*P*=.30) was found on SHEs. This is due to the user’s willingness to exchange aspects of trust in information sources such as the functionality of the platform and familiarity with the platform or the existence of other internal cognitive or psychological aspects that can be investigated in the future.

**Conclusions:**

This study can provide guidance for the development of more effective health communication strategies in the digital era using short video platforms.

## Introduction

### Background

With the advent of social media, access to health information has increased, with more than 55% of adults using social media to seek health information and advice [1]. Vranica and Kruppa [2] found that about 23% of social media users used short video platforms, specifically TikTok. TikTok handles a high search activity, which reached 1.2 million search requests per minute in 2024 [3]. Liu et al [4] explained that the short video-based social media format is most popular for users conducting health information searches because short videos can help convey complex health information in a short time with a combination of visual, audio, and narrative that is easy to understand.

Short video platforms can present information in various forms of media, such as text (captions), images or photos, audio (podcasts), and videos, both long-duration videos and short-duration videos. This multimedia format allows users to see visualizations of the disease they are experiencing so that it is easier to recognize early symptoms and compare their condition with visual examples in videos or photos for reference [5]. The format of information media in the search for health information can be explained through media richness theory (MRT). MRT explains that “richer” media have an effective ability to explain ambiguous issues [6]. Short video platforms can combine visual, audio, text, and live demonstration elements in a short duration. This allows complex health information to be conveyed well and improves audience understanding quickly. In addition to the media aspect, several other factors affect the behavior of searching for health information on short video platforms, such as personalization and interactivity.

Han et al [7] found that TikTok’s algorithm forms an information space that facilitates the discovery of relevant content that encourages exposure and further searches on the platform. This algorithm serves relevant content based on interactions, such as likes and watch time, so that users can find health content without conducting an explicit search [7]. The behavior of seeking health information on short video platforms can also be understood from a psychological perspective, one of which is through the theory of health belief model (HBM). HBM explains that an individual’s decision to engage in the search for health information is influenced by perceived susceptibility (PSU) and perceived severity (PSE) to a health condition [8]. The higher a person’s risk perception, the greater their tendency to seek information through digital platforms, including social media [9,10]. Priyambodo et al [11] showed that users tend to seek explanations when they are worried about the potential risks they could face. This pattern is visible in the realm of skin health, which is visual and easy to observe. Concerns about acne, dark spots, eczema, or other changes in skin conditions make short video platforms the main choice because they are able to present visual demonstrations, real examples, and personal experiences in person [12,13].

As of 2025, Indonesia has a population of 284.4 million people, with internet penetration reaching 80.66% [14]. The most widely used short video platforms in the country are TikTok, Instagram Reels, and YouTube Shorts [14]. With this high level of social media penetration, Thang et al [15] found that many users accessed skin health information through short video platforms. Short video platforms are a popular source used to search for or share dermatological or skincare information, with high average views per video of 1.6 million views on TikTok alone [15]. Thus, social media plays a major role in the beauty discovery phenomenon. Beauty discovery is the process of searching for information or definitions related to beauty, such as information about products, personal care techniques, and beauty standards.

### Skin Health Information Seeking on Social Media

Although they have many uses, short video platforms also have the risk of presenting disinformation. According to Zhang et al [16], the dissemination of information by lay users can lead to widespread misinformation on social media. Other research has shown that the quality of health videos on these platforms is often low. For example, Ming et al [17] found that the proportion of misinformation in certain disease videos on TikTok could reach 41%‐78% of the total content. In their study, Kirkpatrick and Lawrie [18] found that almost all respondents believed that health misinformation was common on TikTok. The absence of special quality evaluation standards for health information on short video platforms is another obstacle to finding health information on short video platforms. The risk of adopting information without further information verification is further exacerbated by the dominance of beauty content in Indonesia, where the representation of bright and smooth skin is a visual standard widely reproduced by content creators [19]. Ariana et al [20] showed that using TikTok significantly affected users’ body image satisfaction. This phenomenon further emphasizes the strong influence of social media on shaping public perception of the definition of an “ideal” body and skin, especially among young audiences who are active in consuming short video content.

Several studies have been conducted on the quality of skin health information content shown on short video platforms. Thang et al [15] discussed acne management information on various short video-based social media platforms and showed that the quality of education varies and does not always correlate with popularity. Khan et al [21] analyzed 120 videos on TikTok related to eczema and found that 65.8% of the content was produced by individuals who did not have a professional background in the health field, while only 15.8% of the content was produced by individuals with a health background. Thang et al [15] found that the quality of content produced by certified dermatologists was significantly higher than that of individual content that did not have a health background. However, videos of better quality made by doctors actually received lower engagement [15]. In addition, several studies have shown misperceptions of information circulating on short video platforms, especially regarding drugs and skin health procedures. Khan et al [21] found many misperceptions about the use of tretinoin, especially exaggerated claims regarding its effectiveness and recommendations for use without medical supervision. A similar pattern can be observed in the content related to derma rolling procedures. For example, Awad et al [22] found that most videos provided recommendations that were not aligned with clinical practice and tended to promise instant results. In general, the most viral videos were those that were demonstrative, emotional, and visually appealing but were not based on strong scientific evidence.

### Objectives

Previous studies have examined the behavior of searching for health information on social media in general [9,11,15,23,24]. However, previous studies have only discussed content quality with regard to the credibility of actual information [21,22,25]. Further research exploring the user behavior of social media platforms with short video formats is needed because skin problems are often related to aesthetic factors, social stigma, and comparison of appearance [26]. Thus, the research question of this study is, “What factors affect the use of short video platforms in searching for skin health information?” The findings of this study can be used by the government, academics, and educational institutions to develop digital literacy and public health programs.

### Conceptual Model

#### Overview

Brzozowska and Gotlib [27] found that positive engagement develops in response to social media, facilitating users’ change behavior on skin health promotion. The stimulus-organism-response (SOR) framework has been used in the health-related studies such as the review study on the impact of social media on skin health promotion [27], analyzing the impact of TikTok on body satisfaction [20], investigating the social media’s impact on sugar reduction behavior [28], and the role of beauty influencer in the digital platforms on impulse buying [29]. Thus, this study adopts the SOR framework to develop a deeper understanding of stimuli originating from short video platforms. Cognitive and perceptual constructs are conceptualized as organism-level processes, while skin health information–seeking behavior serves as the response. Within this framework, stimuli refer to external environmental factors that provoke user responses, whereas the organism reflects users’ internal cognitive processes [29]. User response is defined as the resulting behavioral reaction that emerges from the interaction between these external stimuli and the organism’s internal processes [29]. Therefore, MRT [23] and HBM [9] were adopted in this study to analyze the stimulus and organisms’ internal processes from the social media users for skin health information searching on short video platforms. Grounded in SOR framework, we integrated MRT (eg, content expressiveness and personalization) as stimulus to explain the compatibility of the characteristics of short video media with the visual nature of skin health information, while HBM (eg, perceived usefulness [PU], severity, and susceptibility) as organism-level processes to understand the perceptual and motivational factors of individuals that drive the search for skin health information on short video platforms.

**Figure 1.**
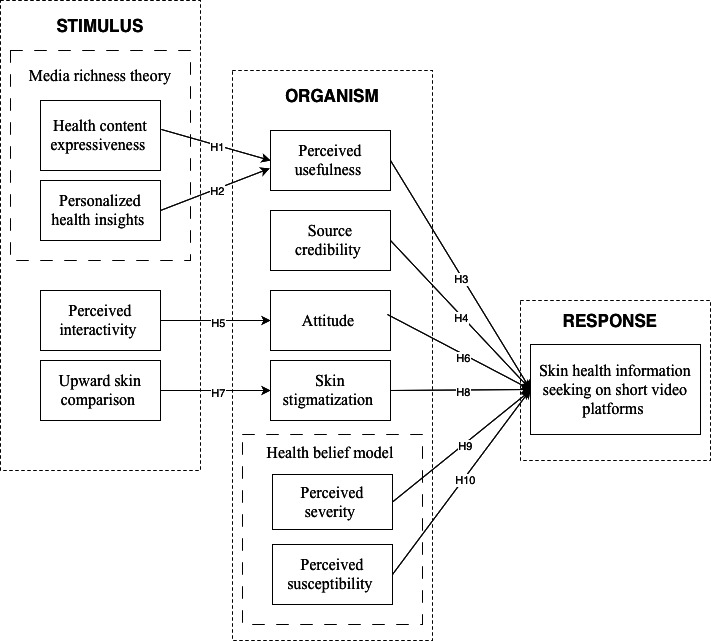
Conceptual model. H: hypothesis.

Yu et al [23] found that media richness affects the PU of a platform, which increases the adoption of health information on the platform and the continued use of the platform in seeking health information. Health content expressiveness (HCE) and personalized health insights (PHIs) were found to have a significant influence on PU [23]. Niu et al [30] revealed that interactivity positively influenced users’ attitudes toward skin health web pages. In addition, Adkins et al [26] found that upward appearance comparisons had a positive influence on feelings of stigmatization in the use of photo-based or visual applications. Malik et al [9] discovered that individual perceptions of a health risk affect users’ desire to seek health information. Figure 1 illustrates the research model proposed in this study.

#### Hypothesis Development

- Hypothesis 1: HCE positively affects PU

HCE is one of the features that contribute to media richness in MRT, which has an influence on media usefulness [23]. Based on MRT, in the context of media richness, HCE is a component that fulfills the aspect of multiple cues, which refers to the use of various communication channels [23]. With increasing media richness, the ambiguity of information will decrease and encourage the positive behavior of information recipients [31]. According to MRT, through various media, such as visual, audio, and text, content expressiveness can enrich the user experience and improve users’ ability to process complex health information [32]. The combination of these various media enables users to have different ways of processing and understanding information [23]. In the context of this study, HCE describes the ability of short video platforms to provide a variety of media, such as visual, audio, and text, which can be used by their users in presenting skin health information. Lenczowski et al [33] found that skin health education in the form of videos was more interesting than written materials. In addition to text, visual and audio elements are important in conveying skin health information online, as skin conditions are visual, and there are many treatment skills, such as self-examination, use of sunscreen, and preparation for actions, that require both visual and verbal demonstrations. If short video platforms can provide all the media needed, then the ability of users to process and adopt the required skin health information will increase as well as increasing user perception of the usefulness of short video platforms in seeking skin health information.

- Hypothesis 2: PHIs positively affect PU

Based on MRT, PHIs are components that meet the aspect of personal focus, which refers to the level of suitability of communication with individual needs [23]. By tailoring recommended health information to the user’s preferences, needs, and context, PHIs can increase the practical value of content, as users perceive the platform to address users’ unique health challenges [23]. In the context of this study, PHIs describe the ability of a short video platform to display skin health information videos that fit the user’s preferences, needs, and context. In addition, personalization has a significant positive influence on PU in the context of mobile health [34]. Personalization can be conducted by using demographic data, health issues, or user interaction history on a platform [23]. Nittas et al [35] found that personalization on educational platforms regarding the use of sunscreen was well received and considered useful by participants. With a recommendation algorithm that captures and learns every action from a user, such as likes, follows, comments, favorites, and input users against the recommendation results, short video platforms have succeeded in increasing users’ use of the platform [36]. Based on MRT, if the short video platform can provide recommendations or display skin health information videos tailored to users’ preferences, needs, and contexts, then users will feel that the short video platform can answer their skin health problems [23]. Personalization is considered an added value in the search for skin health information because people’s skin conditions vary; thus, common skin diseases, such as acne or psoriasis, can have different treatment responses [37]. The proper treatment of a skin condition can require a long trial-and-error process and can frustrate patients; thus, the personalization achieved through virtual experiments increases users’ adoption desires to search skin health information videos on short video platforms, hence, increasing PU of the short video platforms [37].

- Hypothesis 3: PU positively affects skin health information seeking on short video platforms (SHEs)

PU is defined as the level of user confidence that the use of an information system can improve user performance or provide real benefits [23]. Liu et al [4] define PU as a user’s subjective assessment of the value of a short video in guiding users regarding health behaviors. Xia et al [38] found that PU had a significant positive influence on users’ desire to search for health information online, even exceeding the influence of perceived use. In the context of this study, PU describes the user’s perception of the extent to which the short video platform can help users to search and disseminate skin health information. Boyce et al [39] also found that PU had a significant positive influence on health information–seeking behavior in the online health community. In looking for skin health information, one aspect that plays an important role is users’ perceptions of the benefits of a platform in finding relevant and applicable information to solve problems or user needs [40]. With features that make the search process more efficient and relevant, the perception of a platform’s usability can increase [35]. This increased perception of platform usefulness can then encourage users to use a platform to search for skin health information [39].

- Hypothesis 4: SC positively affects SHE

Source credibility (SC) is defined as the perception of the recipient of information regarding the credibility of the source of knowledge or informant [41]. It is a peripheral cue or precursor used before the user decides to engage further with the information provided [42]. In their study, Claggett et al [42] found that users rated the informant as credible and trustworthy when there was a health degree listed on the informant’s name. In seeking health information, the level of credibility of informants was found to have a significant positive influence on the frequency of use of a source to seek information [43]. According to Fan et al [43], sources that are considered credible are more often used by individuals when searching for health information. In the context of short videos such as TikTok, Kirkpatrick and Lawrie [18] found that whistleblowers with professional credentials or markers of affiliation with relevant agencies increased the tendency and frequency of user information searches against those whistleblowers. In the context of skin health, this is because skin health products that are not used appropriately, such as frequency and duration of use, can have side effects on users’ skin. Therefore, the credibility of skin health informants is important to ensure that users are educated with credible information so as to avoid adverse or even harmful side effects; thus, the credibility of the information source can increase the user’s desire to search for information on the source.

- Hypothesis 5: PI positively affects attitude

Perceived interactivity (PI) is defined as the user’s perception of the extent to which the user feels that their experience of using an application resembles interpersonal interaction and provides a sense of social connection [4]. McMillan and Hwang [44] found that PI is influenced by 3 main factors, namely, 2-way communication, user control, and speed or responsiveness. In the context of this study, PI describes the ability of short video platforms to make it easier for their users to interact. Interactions that can be carried out by users do not only include 2-way communication but also include 1-way interactions, such as likes and comments, that allow users to control the videos displayed based on their interactions. Bidirectional communication and user control intersect in active or passive participation factors, user control and speed or responsiveness intersect in interesting or complex activity factors, and speed or responsiveness and bidirectional communication intersect in synchronous or asynchronous communication factors [44]. On TikTok, interactivity was found to be one of the factors contributing to the flow of application use and increasing the desire of users to continue using the application [18]. The interactions made on TikTok, such as likes, comments, and watching content of a longer duration, affect the content recommended by TikTok. Recommended content is tailored to the interaction carried out; thus, user interaction on short video platforms can improve the personalization of the content displayed according to the users’ interests. In the context of skin health, personalization is an added value due to different skin conditions [37]. Therefore, when users can have 1-way or 2-way interactions and feel the platform’s quick or appropriate response to these interactions, their attitude toward a platform tends to improve [45].

- Hypothesis 6: attitude positively affects SHE

Attitude is defined as an individual’s positive or negative feelings toward the use of short videos for health information [4]. Link et al [46] show that attitudes toward seeking health information online, along with risk perception, are the main factors driving individuals’ health information–seeking behavior. Mirzaei et al [47] also found that attitudes represented by user experience are one of the significant predictors of health information search in digital contexts. In the context of this study, attitude describes the user’s feelings toward short video platforms to seek skin health information. In this study, users’ assessments of their experience were influenced by users’ perceptions of the interactivity of short video platforms. Li et al [48] found that users’ positive perceptions and attitudes toward health information are associated with increased health information search behavior in digital services. In the context of social media, Neely et al [49] found that user engagement on social media platforms as well as user attitudes are the driving factors for users’ intentions and behaviors to search for health information on social media platforms. Specifically, on short video platforms, Kirkpatrick and Lawrie [18] found that the intention and behavior of obtaining health information on TikTok had a high percentage, reaching 65.5% (n=672) for users who intentionally searched for health information and 92.4% (n=948) for those who accidentally searched for it. Kirkpatrick and Lawrie [18] also discovered that users have a positive perception of TikTok in the context of searching for health information. Users’ positive attitudes toward online health information searches are associated with higher online health information search behaviors [46]. When users positively rate their experience of searching for health information on short video platforms, users tend to increase the frequency and depth of searching for skin health information through the platform.

- Hypothesis 7: Upward skin comparison (USC) positively affects skin stigmatization (SST)

Adkins et al [26] describe USC as an individual’s tendency to compare the appearance of his or her skin to others who have better skin. USC also has a positive correlation with stigmatization in adults with acne-prone skin conditions [26]. USC is a core process that exacerbates the stigma felt by individuals [26]. Tian et al [50] also found that upward social comparisons on social media increase an individual’s anxiety about his or her appearance, which is closely related to the individual’s stigma and psychosocial burden. In the context of dermatology, Van Beugen et al [51] found that patients with skin diseases that can be seen without aids experienced higher stigmatization than other patients. This suggests that skin conditions that can be seen without aids tend to encourage users to compare themselves, thus creating a psychosocial burden. In the context of this study, USC is defined as an individual’s tendency to compare the condition or appearance of their skin with other individuals who are rated as having “better” skin. This tendency is not only limited when individuals use short video platforms only but also the tendency to compare in everyday life. However, the use of social media, especially short video platforms with visual dominance and “ideal skin” content, also contributes by increasing the possibility for individuals to compare their skin conditions with those of other creators or users [26]. Adkins et al [26] found that individuals who compared their skin conditions with other people’s skin conditions had higher feelings of stigmatization. Tunca et al [52] found that patients with acne vulgaris disease have significant physical manifestations and emotional and social implications. The disease can increase feelings of guilt, shame, social anxiety, and social isolation [52]. Tunca et al [52] also found that patients with acne often found themselves less attractive, could experience withdrawal behaviors, and avoided social gatherings for fear of being judged.

- Hypothesis 8: SST positively affects SHE

SST is defined as self-perception and anticipation of skin-related social judgments associated with an individual’s internalized negative feelings due to certain skin conditions, such as acne [26]. Berger et al [53] revealed that individuals with conditions who experienced stigmatization because of their disease were found to use the internet more often to seek health information than individuals who did not experience stigma. In the context of skin health, Aslan Kayıran et al [54] found that patients with psoriasis routinely use social media and the internet to find information about their disease. This further strengthens the finding that digital platforms are one of the digital channels used by individuals to seek skin health information. Nguyen et al [55] discovered that social media platforms are a relevant and widely used dermatology reference source for patients seeking skin health information. This is due to the accessibility of information, the ease of use of social media platforms, and the social characteristics of the platform [56]. In the context of this study, SST is defined as individuals’ negative perceptions of themselves caused by their skin conditions. Negative perceptions or the way individuals judge themselves are not only limited to the clinical severity of the skin condition but also include the anticipation of rejection, sensitivity to the attitudes of others, shyness, and a tendency to hide their skin conditions, which is mapped in the feelings of stigmatization instrument for dermatology patients [57]. In addition to internal factors, these judgments are shaped by social behaviors, such as stereotypes, prejudices, and discrimination [58]. Tunca et al [52] found that skin conditions have physical and emotional effects on patients with certain skin conditions that can be seen without aids, such as acne and psoriasis. The emotional impact experienced can be in the form of feelings of guilt, shame, anxiety, and social isolation [52]. Rowlands et al [59] found that individuals with health conditions who are prone to stigma tend to seek health information through digital platforms. This is because the stigma possessed by individuals encourages them to avoid formal help and choose to look for alternatives, such as social media platforms [58].

- Hypothesis 9: PSE positively affects SHE

Based on HBM, PSE refers to an individual’s belief in the intensity of a medical condition and the seriousness of consequences related to a particular health problem [9]. Malik et al [9] found that PSE has a positive influence on individuals’ willingness to search for health information through social media. When PSE increases, individuals assess health threats as greater so that the benefits of searching for health information outweigh the effort needed to conduct the search [10]. In addition, in the context of social media, higher PSE encourages users to seek explanations, guidance, and action options that can be done through quick and easily accessible platforms, such as social media platforms [60]. Neely et al [49] also found that during risky public health situations, users with high PSE conducted more searches for health information through social media to reduce uncertainty and guide health decision-making. Moreover, patients with serious skin conditions routinely use the internet to search for skin care information [54]. Based on HBM, when individuals assess their skin conditions as serious, such as potentially interfering with social interactions, self-confidence, and daily activities, these perceptions can be a driving factor for individuals to seek skin health information as a protective tool that can help them and others avoid or reduce the risks that arise from certain skin health problems [9].

- Hypothesis 10: PSU positively affects SHE

Based on HBM, PSU is defined as an individual’s belief about the possibility of being affected by a health problem [9]. PSU is one of the significant predictors of the behavior of seeking online health information [10,46]. When individuals feel vulnerable to a health problem, they have a tendency to seek information on ways to reduce the risk [61]. Therefore, when PSU increases, the expected benefits are considered higher than the effort required, so that individuals are motivated to search for information. In the context of this study, PSU is defined as an individual’s perception of the likelihood of being affected by certain skin health problems. This possibility is not only assessed against infectious diseases but also against possibilities caused by genetic factors or family history and nongenetic factors, such as infections, use of certain medications, lifestyle, and the environment. Richard et al [62] found that the location of certain diseases has a different impact on the patient’s daily life, and this is due to the burden of the visibility of a disease. Moreover, facial perception and visual attention directed at the face and hands are essential in human social communication; thus, diseases in these parts have a more significant effect [62]. Based on HBM, an increase in PSU strengthens perceived threats, which are predictors of online health information–seeking behavior [9,10]. Perceived threats are also predictors of information insufficiency, which increases the intention to seek information [63]. In the context of social media platforms, users with higher PSU were found to search for health information more actively than users with lower PSU [49].

## Methods

### Ethical Considerations

This study was approved by the Faculty of Computer Science, University of Indonesia (reference S-20A/UN2.F11.D1.5/PPM.00.00/2025 and dated June 3, 2025). All of the respondents involved in this study agreed in writing to participate in the study, and all respondent data were anonymized. The questionnaire was filled in by the respondents voluntarily, and they could withdraw from participating in the research at any time. We did not provide incentives for the respondents. We used the survey tools of the University of Indonesia and stored the data exclusively on the university’s server. All respondents approved the written consent to be involved in this study.

### Study Design

This study adopted a mixed methods approach, using an online questionnaire (quantitative) and interviews (qualitative). We designed an online and open questionnaire based on the CHERRIES (Checklist for Reporting Results of Internet E-Surveys) checklist (Checklist 1). All respondents approved the written consent to be involved in this study. The respondents were selected according to the criteria of the research target—that is, Indonesian people who used short video platforms to search for skin health information (purposive sampling). The questionnaire link was shared for 28 days from September 22, 2025, until October 20, 2025, through social media platforms widely used by Indonesian people, such as Facebook, Instagram, and TikTok. In the early stages, quantitative research was used as the primary method to test the hypotheses and the relationships between the variables in the research models. Furthermore, a qualitative approach was carried out to explain and enrich the understanding of the quantitative results through interviews with 30 respondents.

### Research Instruments

The research instrument was in the form of a questionnaire, which was divided into 3 pages: respondent validity, respondent demographics, and 49 measurement items (Multimedia Appendix 1). The questionnaire completion time was 10‐15 minutes, and the respondents could review their answers by clicking the back button for each page. We did not randomize the questionnaire’s statements and did not use adaptive questions. For mandatory questions and statements in the questionnaire, we put a red star where the respondents must fill in the questions and statements before they could click the submit button. The respondent validity section consists of questions regarding the knowledge and experience of using one of the short video platforms used by the user, that is—TikTok, Instagram Reels, or YouTube Shorts. In addition, the respondent demographics section consists of questions related to the background of the study respondents. The measurement items were measured using a 5-point Likert scale, in which a value of 1 was interpreted as “strongly disagree,” and a value of 5 was interpreted as “strongly agree.”

### Questionnaire Measurement Item Construction and Pretest

The readability test was conducted to evaluate the ease of the questionnaire questions and to identify aspects that needed improvement from the respondents’ perspectives. The readability test was carried out in the form of interviews with 13 respondents. The respondents provided input on the right word selection and the correction of word or punctuation errors. After the readability test stage was completed, the pilot study stage was conducted with 30 respondents to test the reliability of the research indicators that had been made previously. The total Cronbach α (CA) score for the results of the pilot study was 0.928; thus, the questionnaire could be disseminated to a wider range of respondents. Multimedia Appendix 2 presents the demographics of the 603 valid respondents.

### Data Cleansing

Of the 889-questionnaire data received, we conducted a manual data completeness check using Microsoft Excel to ensure that all questions had been answered completely. An examination of the results showed that 245 respondents did not complete the questionnaire until the end; thus, the data from these respondents were unable to be used for hypothesis testing. The remaining 644 questionnaires were completely filled out for hypothesis testing.

### Analysis Methods

Questionnaire data were processed using the covariance-based structural equation modeling method using AMOS SPSS (version 26; IBM Corp) software, which aims to test the relationships between the variables formulated in the research model. This method was chosen because the study focused on testing hypotheses based on theories that had been tested in previous research. Qualitative data analysis was conducted using thematic analysis. Thematic analysis was chosen because it provides flexibility in identifying, analyzing, and interpreting patterns or themes that systematically emerge from qualitative data [64]. The social, cultural, and psychological contexts behind user behavior can also be found using thematic analysis [64].

## Results

### Common Method Bias

To detect potential bias, one of the approaches carried out was the confirmatory factor analysis (CFA) single-factor test, which determines one common latent factor that contains all the research indicators [65]. This approach is conducted by estimating the CFA model by pointing all indicators to one common factor as a form of testing whether one common factor can explain most of the variance of the data [65]. Common method bias (CMB) testing using single-factor CFA can be performed by analyzing the feasibility of the model [66]. If the single-factor model does not have a good goodness of fit (GoF), then CMB is not identified in the research model, and the research can continue to the next stage [66]. Table 1 describes the CMB results for measurement and structural model testing.

**Table 1.** Common method bias results.

GoF^a^ metrics	GoF criteria		Common factor values	Description
	Good fit	Acceptable fit		
CMIN/df^b^	≤2	≤3	7.526	Poor fit
GFI^c^	≥0.95	≥0.9	0.387	Poor fit
RMR^d^	≤0.05	≤0.1	0.109	Poor fit
NFI^e^	≥0.95	≥0.9	0.439	Poor fit
CFI^f^	≥0.97	≥0.9	0.420	Poor fit
TLI^g^	≥0.97	≥0.9	0.449	Poor fit
RMSEA^h^	≤0.05	≤0.08	0.127	Poor fit

a GoF: goodness of fit.

b CMIN/df: minimum discrepancy of confirmatory factor analysis/degrees of freedom.

c GFI: goodness of fit index.

d RMR: root mean square residual.

e NFI: normed fit index.

f CFI: comparative fit index.

g TLI: Tucker-Lewis index.

h RMSEA: root mean square error of approximation.

### Measurement and Structural Model Testing

The convergent validity test was carried out by analyzing the average extracted variance (AVE) value, in which the minimum AVE value for each variable tested must be above 0.5 (Hair et al, 2019). As shown in Table 2, all variables met the AVE value ≥0.5. Therefore, the convergent validity test stage was completed, and the study continued to the next stage. The reliability of the construct was measured using CA and composite reliability. Acceptable CA and composite reliability values are greater than 0.70 [67].

**Table 2.** AVE^a^, CA^b^, and CR^c^ values.

Variable	AVE	CA	CR
AT^d^	0.653	0.883	0.882
HCE^e^	0.613	0.864	0.863
PHI^f^	0.593	0.814	0.813
PI^g^	0.685	0.867	0.867
PSE^h^	0.658	0.852	0.852
PSU^i^	0.606	0.885	0.885
PU^j^	0.681	0.914	0.914
SC^k^	0.677	0.913	0.913
SHE^l^	0.727	0.930	0.930
SST^m^	0.702	0.904	0.904
USC^n^	0.641	0.877	0.877

a AVE: average extracted variance.

b CA: Cronbach α.

c CR: composite reliability.

d AT: attitude.

e HCE: health content expressiveness.

f PHI: personalized health insight.

g PI: perceived interactivity.

h PSE: perceived severity.

i PSU: perceived susceptibility.

j PU: perceived usefulness.

k SC: source credibility.

l SHE: skin health information seeking on short video .

m SST: skin stigmatization.

n USC: upward skin comparison.

The discriminant validity test was performed to ensure that each construct measured in the research model had a significant difference from the other constructs [67]. This stage was conducted to ensure that a construct not only measures the same concept as other constructs but also has unique characteristics so that it has to be separated as a construct in the research model [67]. Table 3 presents the results of the discrimination validity test.

**Table 3.** Correlation matrices.

	PSU^a^	PSE^b^	USC^c^	PI^d^	SC^e^	PHI^f^	HCE^g^	AT^h^	SST^i^	PU^j^	SHE^k^
PSU	0.494										
PSE	0.321	0.470									
USC	0.267	0.264	0.495								
PI	0.131	0.144	0.176	0.366							
SC	0.137	0.135	0.134	0.213	0.364						
PHI	0.082	0.125	0.153	0.227	0.189	0.307					
HCE	0.096	0.128	0.111	0.204	0.131	0.148	0.414				
AT	0.096	0.106	0.129	0.269	0.156	0.167	0.150	0.340			
SST	0.198	0.196	0.366	0.130	0.099	0.113	0.082	0.095	0.624		
PU	0.078	0.113	0.122	0.195	0.149	0.219	0.228	0.143	0.091	0.344	
SHE	0.165	0.190	0.168	0.223	0.165	0.182	0.178	0.222	0.126	0.215	0.398

a PSU: perceived susceptibility.

b PSE: perceived severity.

c USC: upward skin comparison.

d PI: perceived interactivity.

e SC: source credibility.

f PHI: personalized health insight.

g HCE: health content expressiveness.

h AT: attitude.

i SST: skin stigmatization.

j PU: perceived usefulness.

k SHE: skin health information seeking on short video platform.

GoF testing was performed to evaluate the feasibility of the research model by determining whether the GoF values met the specified criteria [67]. The evaluation was carried out by evaluating several GoF values, such as the relative chi-square, GoF index, root mean square residual, normal fit index, comparative fit index, Tucker-Lewis index, and root mean square error of approximation [67]. Table 4 presents the GoF values of the research model.

**Table 4.** Goodness of fit (GoF) value.

GoF metrics	GoF criteria	Results
CMIN/df^a^	≤2	1.242
GFI^b^	≥0.9	0.906
RMR^c^	≤0.1	0.032
NFI^d^	≥0.9	0.930
CFI^e^	≥0.9	0.985
TLI^f^	≥0.9	0.982
RMSEA^g^	≤0.08	0.025

a CMIN/df: minimum discrepancy of confirmatory factor analysis/degrees of freedom.

b GFI: goodness of fit index.

c RMR: root mean square residual.

d NFI: normed fit index.

e CFI: comparative fit index.

f TLI: Tucker-Lewis index.

g RMSEA: root mean square error of approximation.

### Hypothesis Testing

Hypothesis testing was performed using a 1-tailed test to assess the significance of the evaluated hypothesis coefficient. A hypothesis was declared acceptable if the *P* value <.05, while the hypothesis was rejected if the *P* value was ≥.05 [67]. The results of the hypothesis test are summarized in Table 5, which shows that out of the 10 hypotheses submitted, 8 hypotheses were accepted, and 2 hypotheses were rejected because they had a *P* value ≥.05.

**Table 5.** Hypothesis (H) test results.

Hypothesis				Estimate	*P* value	Results
H1: PU^a^←HCE^b^				0.425	.001	Accepted
H2: PU←PHI^c^				0.491	<.001	Accepted
H3: SHE^d^←PU				0.327	.01	Accepted
H4: SHE←SC^e^				0.063	.17	Rejected
H5: AT^f^←PI^g^				0.754	<.001	Accepted
H6: SHE←AT				0.302	.02	Accepted
H7: SST^h^←USC^i^				0.627	<.001	Accepted
H8: SHE←SST				−0.041	.30	Rejected
H9: SHE←PSE^j^				0.161	.009	Accepted
H10: SHE←PSU^k^				0.159	.02	Accepted

a PU: perceived usefulness.

b HCE: health content expressiveness.

c PHI: personalized health insight.

d SHE: skin health information seeking on short video platform.

e SC: source credibility.

f AT: attitude.

g PI: perceived interactivity.

h SST: skin stigmatization.

i USC: upward skin comparison.

j PSE: perceived severity.

k PSU: perceived susceptibility.

Based on the results of the effect size analysis, it can be seen that each construct in the model has a different influence power. The attitude variable showed an effect size of 0.569, which was in the range of 0.50‐0.79 so it was included in the category of medium effect. These findings show that PI has a strong and substantive influence in shaping users’ attitudes. Furthermore, the SST variable has an effect size of 0.392, which is in the category of small effects, so although USC has an effect on increasing skin stigma, the power of influence is relatively weaker than other constructs. The PU variable showed an effect size of 0.562, so it was also included in the medium effect category. This indicates that the combination of the influence of HCE and PHIs makes a significant contribution in increasing the perception of the usefulness of skin health content. Meanwhile, the variable SHE has an effect size of 0.579, which is also in the medium category. This shows that related variables in the research model, such as PU, attitude, PSE, and PSU, have a strong contribution in encouraging skin health information–seeking behavior on short video platforms.

## Discussion

### Summary of the Findings

The study found that HCE and PHIs affected users’ PU of short video platforms in searching for skin health information, thus affecting SHEs. SC had no effect on SHEs because the functionality of the platform and familiarity with the platform were the main points chosen by users. PI had an effect on attitudes, thus affecting SHEs. In the context of short video platforms, interactivity is one of the factors that play a significant role because it is considered to increase personalization, which was previously found to have a positive influence on users’ feelings on a platform. USC also had an effect on SST, which had no effect on SHEs. Finally, PSE and PSU affected SHEs.

All qualitative results in each hypothesis strengthen the quantitative results and provide the detailed reason of each hypothesis results according to the Indonesian context. Table 6 describes the mapping summary of the hypothesis testing results with the qualitative results. The unique findings of this study were on the results of SC and SST that has no effect on SHEs. SC only affected an individual’s desire to conduct further searches to validate the information they have just watched. Trusting a figure was also conducted one by one when the content was being watched and not before doing a search and through the dynamics of interactions in the platform ecosystem. Furthermore, in the context of users of short video platforms in Indonesia, SST is not the main factor that drives the behavior of seeking skin health information, especially on short video platforms. The next section will discuss further justification of each hypothesis.

**Table 6.** Mapping summary of the hypothesis (H) testing results with the qualitative results.

Hypothesis	Hypothesis testing results	Summary of the qualitative results
H1	Accepted	The expressiveness of the content on the short video platform provides visual evidence, makes it easier to understand, clarifies how to use the product, and presents a direct picture of skin conditions, thus making skin health information more useful and relevant for users.
H2	Accepted	Personalization allows users to receive content relevant to their personal needs, expand insights through additional recommendations, improve search efficiency, and simplify the process of finding skin health information. While some concerns have been raised regarding privacy and information overload, personalization is still perceived as a feature that can improve the usefulness of skin health information on short video platforms.
H3	Accepted	Perceived usefulness is an important factor that encourages users to search for skin health information on short video platforms through ease of access, information relevance, interactivity, and the platform’s ability to provide additional insights.
H4	Rejected	The credibility of informants remains important, but perceptions of the level of credibility do not affect an individual’s desire not to conduct searches on short video platforms. Rather, they affect an individual’s desire to conduct further searches to validate the information they have just watched.
H5	Accepted	Interactions on short video platforms through like, comment, and save features make users feel that they have appreciated content creators who provide useful skin health information. This interaction also positively affects user attitudes because users feel engaged and have control over the videos displayed on the short video platform.
H6	Accepted	In the context of searching for skin health information on short video platforms, attitudes can be judged through users’ feelings toward short video platforms to search for skin health information. With the positive feelings of users toward the process of searching for skin health information on short video platforms, users have a desire to search for health information on short video platforms.
H7	Accepted	Users feel motivated to have as good a skin condition as that shown on the short video platform, are more diligent in taking care of their skin, and strive to solve the problems experienced. Users can also understand the content displayed using filters and makeup and promotional content in the form of marketing, which is not actually used by the content creator.
H8	Rejected	Most of the interviewees did not feel stigmatized and were used to their skin conditions; thus, the stigma did not trigger the search for health information. Even when users’ confidence in their skin health increases, some prefer to seek information from other sources (eg, doctors, clinics, or buying products directly) rather than using short video platforms. Thus, in the context of users of short video platforms in Indonesia, skin stigmatization is not the main factor that drives the behavior of seeking skin health information, especially on short video platforms.
H9	Accepted	The higher the perceived severity, the greater the individual’s tendency to have skin health information–seeking behavior on short video platforms. Concerns about potential long-term effects and a desire to avoid or minimize skin problems are the main factors that drive users’ search for information, both as preventive and early mitigation measures before deciding to seek professional help.
H10	Accepted	The higher the individual’s concern about the possibility of skin problems, the greater their tendency to be actively involved in the search, verification, and application of skin health information through short video platforms.

### The Effect of HCE on PU

The study found that HCE significantly affected PU. These findings are consistent with Yu et al [23], Armstrong et al [68], and Lenczowski et al [33]. Yu et al [23] found that the use of media elements, such as visual, audio, and text, could enrich the user experience and improve the understanding of complex health information. Armstrong et al [68] revealed that skin health education through online videos was more effective than written media because videos allow for live demonstrations. Lenczowski et al [33] showed that their respondents preferred skin health education through video rather than written materials, confirming that expressive media improves the perception of the usefulness of information.

The findings of the qualitative research also support the results of the quantitative analysis (hypothesis 1). In total, 9 interviewees (9/30, 30%) stated that the expressiveness of content on short video platforms helped them to see first-hand evidence when searching for skin health information. Interviewee 15 said:

*I can see the results of using the product, my skin texture, and how to use it directly. Of all visual media, photos and videos are important because they provide a real picture and make it easier for me to judge the product or the results*.

In addition, 13 interviewees (13/30, 43.33%) felt that the expressiveness of content on short video platforms made it easier for them to capture information. Interviewee 9 stated:

*The media is very helpful to see visualizations and make information easier to understand. So, not only do you hear the explanation, but there is also a picture. The most important thing is still videos because you need to see the visuals directly*.

A total of 7 interviewees (7/30, 23.33%) also found this helpful because the expressiveness of short video content on short video platforms can show how the product is used. As Interviewee 22 explained:

*Visuals are very helpful in giving an overview of the product measurements. If you just write about it, it’s usually less imaginative. For example, in the past, I was confused about how much moisturizer to use because each person has a different finger size. The video makes the measurement clearer, so the video is the most important.*

The expressiveness of the content on the short video platform provides visual evidence, makes the content easier to understand, clarifies how to use the product, and presents actual pictures of skin conditions, thus making skin health information more useful and relevant for users.

### The Effect of PHIs on PU

This study found that PHIs had a significant effect on PU. The results of this study are in accordance with Yu et al [23], Zhang et al [34], and Nittas et al [35]. Yu et al [23] found that PHIs are one of the features of media richness that significantly affect PU on short video platforms. Information that is appropriate to the users’ preferences, needs, and context provides the content with more practical value and can effectively address individual health challenges [23]. Zhang et al [34] showed that PHIs in the context of mobile health had a positive and significant influence on PU because they tailored services to the unique conditions of users in China. Nittas et al [35] found that personalization of skin health education, such as the use of sunscreen, was well received by participants in Switzerland and improved their perception of the platform’s usability.

The results of the interviews support the findings from the questionnaire results (hypothesis 2). In total, 9 interviewees (n=30, 30%) stated that personalization helped them obtain skin health information according to their needs. As Interviewee 8 mentioned, “Personalization makes it easier because the content that appears is already relevant to the question or skin condition that is being experienced.” Personalization in the context of skin health on short video platforms is able to display content that is aligned with users’ individual dermatological needs based on search history, duration of viewing, interactions, and user preferences for certain skin types or skin concerns. In addition, 7 interviewees (n=30, 23.33%) felt that personalization helped them obtain more information related to skin health. Interviewee 3 said:

*Personalization makes it easy for me to get information from various sources. I still need varied content to get insights from many sides*.

Personalization allows users to receive content relevant to their personal needs, expand insights through additional recommendations, improve search efficiency, and simplify the process of finding skin health information. While some concerns have been raised regarding privacy and information overload, personalization is still perceived as a feature that can improve the usefulness of skin health information on short video platforms.

### The Effect of PU on SHEs

PU had a significant effect on SHEs. The results of the quantitative analysis are in accordance with Yu et al [23], Liu et al [4], Xia et al [38], and Boyce et al [39]. Yu et al [23] showed that PU is one of the main factors driving the search for health information on short video platforms, as users assess the content provided as useful and can improve their ability to understand health information. Xia et al [38] found that PU had a significant positive influence on users’ desire to search for health information online, even greater than the effect of perceived ease of use. In addition, Boyce et al [39] emphasized that the PU of a platform drives the behavior of seeking health information in online communities because users feel that the platform helps them find appropriate and relevant information.

In addition, we found that the interview results support the questionnaire result (hypothesis 3). Based on the interviews, 3 interviewees (n=30, 10%) considered short video platforms beneficial because they supported discussion and interactivity, which facilitated the process of finding and understanding skin health information. Interviewee 12 stated, “TikTok remains the main platform for me because its features are interactive and make it easier for me to understand information quickly.” Aside from interactivity, the perception of usability was also reflected in the ease of the search process. A total of 9 interviewees (n=30, 30%) stated that short video platforms offer a fast, practical, and easy-to-understand search process. As explained by Interviewee 13, “Because the content has been adjusted to my needs, the information that appears becomes relevant and quickly connects with what I’m looking for.” The benefits of the platform were also seen through the platform’s ability to provide additional information beyond the users’ initial needs. In total, 6 interviewees (n=30, 20%) revealed that short video platforms helped broaden their horizons. As stated by Interviewee 1, “Sometimes I get additional information that I didn’t think of before.” Aside from adding insights, other interviewees emphasized that the platform also provided a variety of information and the latest content that has been curated. Therefore, PU is an important factor that encourages users to search for skin health information on short video platforms through ease of access, information relevance, interactivity, and the platform’s ability to provide additional insights.

### The Effect of SC on SHEs

SC did not have a significant effect on SHEs. Moreover, the results of this study are not in accordance with those of Fan et al [43] and Kirkpatrick and Lawrie [18]. Fan et al [43] and Kirkpatrick and Lawrie [18] found that assessments of whistleblower credibility can affect the tendency and frequency of user information searches. However, several studies have found that the credibility of information sources did not have a significant effect on the search for health information through online platforms [69,70].

Denniss et al [70] revealed that the credibility of sources was considered less relevant because users judged the content and social interactions more. In the context of the use of short video platforms in the search for skin health information, SC did not affect the search because the characteristics of the social media ecosystem were more content-oriented and algorithmic. In the interviews, the interviewees who were laymen in the context of skin health watched the videos shown by short video platforms but did not specifically search for a figure. Therefore, trusting a figure was carried out one by one when the content was being watched, and this was not done before conducting a search. This is consistent with the demographic distribution of the study, in which 405 respondents (n=644, 67.1%) stated that skincare products were the type of skin health information they were looking for on short video platforms. This type of information is more general; thus, it can be reviewed by anyone without a formal background in the health field. The type of information that anyone can review emphasizes the role of platforms in building algorithmic ecosystems as a key factor compared with the role of individual content creators in the process of searching for skin health information. Trusting a figure was also conducted one by one when the content was being watched and not before doing a search. To date, users also face an increasing number of hoax content, algorithmic contents, and content generated by artificial intelligence [71,72]; thus, making social media users more careful in looking for informational or educational contents especially for “algorithmic truth” hindering. Figures should align their core values by emphasizing quality and environmental sustainability in their messaging that can strengthen users’ trust and emotional connection in the short video platform [73]. Moreover, quantity and quality social proof (ie, likes and comments) could also influence users’ behavior in the short video platform [74].

Moreover, interview results also support the quantitative result (hypothesis 4). We found that 20 interviewees (n=30, 67.1%) revealed that skincare products were the type of skin health information they were looking for on short video platforms. This type of information is more general; thus, it can be reviewed by anyone without a formal background in the health field. In addition, based on the results of the interviews, the validation of skin health information on short video platforms is more often done through social interactivity mechanisms, such as comments, the number of likes, and follow-up responses in the form of replies or stitch content. These interactions serve as a form of collective evaluation that helps users assess whether information is trustworthy. In this context, credibility is not built individually through a specific figure but is done every time one watches a video communally through the dynamics of interactions in the platform ecosystem.

Trusting a figure was also conducted one by one when the content was being watched and not before doing a search. Therefore, several exchange rates on the platform could make users continue to use short video platforms to search for skin health information. A total of 12 interviewees (n=30, 40%) stated that their trust in creators’ content on short video platforms was quite low, but they still conducted searches on short video platforms. According to Interviewee 27:

*I don’t immediately believe the info from TikTok, anyway. Usually, I dig again and verify it with other content. But if the way of speaking is honest and the form is of an honest review, then it will make me believe more. I also look at the track record, including discussions in the comment column.*

These findings suggest that the credibility of informants remains important, but perceptions of the level of credibility do not affect an individual’s desire not to conduct searches on short video platforms. Rather, it affects an individual’s desire to conduct further searches to validate the information they have just watched.

### The Effect of PI on Users’ Attitudes

PI had a significant influence on attitude. This result is in accordance with Liu et al [4], who found that interactivity is one of the factors that contribute to the flow of application use and increases the desire of users to continue using the application. The finding is also consistent with Haykal [37], who found that personalization is an added value in the context of skin health, as everyone’s skin condition is different. The qualitative results also support the findings of the questionnaire result (hypothesis 5). A total of 6 interviewees (n=30, 20%) revealed that interactions on short video platforms gave them positive feelings because they helped providing personalized information. Interviewee 2 stated:

*I usually like or save if the video is useful or interesting. I rarely comment. During that interaction, I felt satisfied because the FYP became more personal and catered to my needs. So, later, similar content will appear more often*.

In addition, 7 interviewees (n=30, 23.33%) had positive feelings about the interactions because they could help retain information. As Interviewee 7 said, “With the save feature, I now have a kind of personal archive that I can open again at any time without having to search from scratch.” In total, 3 interviewees (n=30, 10%) also had positive feelings about the interactions because the comments showed communal support. According to Interviewee 16, “If my comment is replied to or someone responds, I feel more confident and don’t feel alone.” For 3 interviewees (n=30, 10%), the interactions provided positive feelings because they increased their sense of involvement. Interviewee 15 stated, “When I like or save skin health content, I feel more involved with the content.” Thus, interactions on short video platforms through the like, comment, and save features make users feel that they have shown appreciation to content creators who provide useful skin health information. This interaction also positively affects user attitudes because users feel engaged and have control over the videos displayed on the short video platform.

### The Effect of Users’ Attitudes on SHEs

Attitude had a weak effect on SHEs. This result is in accordance with Li et al [48], Neely et al [49], and Link et al [46]. Li et al [48] showed that users’ perceptions and positive attitudes in China were associated with increased health information search behavior in digital services. Neely et al [49] found that user engagement on social media platforms and user attitudes were factors driving users’ intentions and behaviors to search for health information on social media platforms in the United States. Furthermore, Link et al [46] explained that users’ positive attitudes toward online health information searches were associated with increased online health information search behaviors in Germany.

Moreover, interview results support the findings of the quantitative results (hypothesis 6). We found that 10 interviewees (n=30, 33.33%) felt that using short video platforms helped them search for skin health information; thus, they were encouraged to use short video platforms when they wanted to find skin health information. According to Interviewee 9:

*Yes, I felt that I was given help because my questions were answered. If you need more info, just open the application again*.

Moreover, 8 interviewees (n=30, 26.66%) were satisfied with the experience of searching for skin health information on short video platforms; thus, they were encouraged to use the platform again. As Interviewee 10 stated:

*I’m satisfied so far because the information is complete and can be seen from many angles. This sense of satisfaction makes me continue to use Instagram to look for information*.

In addition, 3 interviewees (n=30, 10%) felt comfortable using the short video platform when searching for skin health information; thus, they were encouraged to use the platform again. As Interviewee 13 revealed:

*I found it helpful because the content was diverse and I could search for a lot of things in one place. This positive feeling made me continue to search for information on TikTok, including about skin health. Because it’s comfortable, I keep it as my main platform*.

Thus, in the context of searching for skin health information on short video platforms, attitudes can be judged through users’ feelings toward short video platforms when searching for skin health information. With users’ positive feelings toward the process of searching for skin health information on short video platforms, they have the desire to search for health information on short video platforms.

### The Effect of USC on SST

USC affected SST. This result is in accordance with Tian et al [50], who found that USCs, particularly on social media, increased individuals’ anxiety about their appearance in the context of general appearance, not specific to the skin. In the context of searching for skin health information on short video platforms, USC is defined as a situation in which users compare their skin conditions with those on content that displays better skin conditions. Our interview results also support the quantitative finding (hypothesis 7). This study found that 3 interviewees (n=30, 10%) admitted that they had compared their skin conditions with those on the short video platform and that this comparison process gave rise to feelings of insecurity. According to Interviewee 3:

*Yes, I used to compare a lot. Now, it’s not too much because I already understand that the breakout phase comes and goes. In the past, I was also insecure, especially when I saw people with smooth skin. I often think about whether my skin would look good if it wasn’t such a mess*.

In addition, 14 interviewees (n=30, 46.66%) were motivated to have a good skin condition similar to that shown on the short video platform, were more diligent in taking care of their skin, and strived to solve the problems they experienced. Interviewee 27 said:

*If you look at the video of people with really good skin, yes, I compare. Sometimes it’s a good idea to take care of your skin more*.

Users can also understand the content displayed using filters and makeup and promotional content in the form of marketing, which is not actually used by the content creator.

### The Effect of SST on SHEs

SST had no effect on SHEs. This finding is not consistent with those of Berger et al [53], Aslan Kayıran et al [54], and Rowlands et al [59]. Berger et al [53] showed that stigmatization due to skin health conditions had a significant effect on the behavior of seeking health information online, as individuals who feel stigmatized tend to look for alternative information through the internet as a form of coping with negative social judgments. Conversely, Aslan Kayıran et al [54] found that the perception of stigma in patients with psoriasis had a significant effect on the search for health information through social media and the internet. Rowlands et al [59] also discovered that SST can encourage individuals to search for health information digitally, as they tend to avoid formal help and opt for private channels, such as online platforms.

In accordance with the quantitative results (hypothesis 8), 6 interviewees (n=30, 20%) did not feel insecure about their skin conditions, as explained by Interviewee 12:

*I’ve never felt uncomfortable or lacked confidence in my skin condition. So far, I’m pretty confident*.

A total of 4 interviewees (n=30, 13.33%) were used to their skin conditions and thus no longer felt insecure. They conducted information searches more as a preventive measure or practical need. Interviewee 21 stated:

*I used to lack confidence, but now I feel normal because I already know the pattern. The hormonal acne will go away on its own*.

According to 2 interviewees (n=30, 6.66%), when they felt insecure about their skin conditions, they would conduct an information search through the help of medical professionals, such as doctors or clinics, rather than on short video platforms. Interviewee 18 said, “When I feel uncomfortable or have a breakout, I will immediately stop using the product and look for a solution directly at a physical place or clinic.” A total of 5 interviewees (n=30, 16.66%) immediately sought and bought skin health products when they felt insecure about their skin conditions. As Interviewee 5 explained, “If I’m insecure because of severe acne, I usually immediately look for products that can help.” Most of the interviewees did not feel stigmatized or were used to their skin conditions; thus, the stigma did not trigger the search for health information. Even when users’ confidence in their skin health increases, some prefer to seek information from other sources (doctors, clinics, or buying products directly) rather than using short video platforms. Thus, in the context of users of short video platforms in Indonesia, SST is not the main factor that drives the behavior of seeking skin health information, especially on short video platforms. Hussain et al [75] found that many women relied on filters for social validation despite recognizing their artificiality; thus, to date, beauty standards are increasingly coproduced by platform design and artificial intelligence–driven filters. In addition, beauty standards also influenced by culture, norm, and value [75].

### The Effect of PSE on SHEs

PSE had a significant effect on skin health information seeking. This result is in accordance with Malik et al [9], Zhao et al [10], and Neely et al [49]. Zhao et al [10] showed that PSE had a positive effect on individuals’ willingness to search for health information through social media in individuals with chronic diseases in China. Increasing PSE was found to increase health information search behavior, as users want to avoid greater health threats [10]. This study is consistent with the study by Neely et al [49], which found that users with high PSE tended to search for more health information to reduce uncertainty in health decisions. Malik et al [9] also asserted that this perception is used to help avoid or reduce the risks that can arise due to certain skin health problems in Pakistan. In the context of searching for skin health information on short video platforms, PSE can be visualized in the content shown on short video platforms.

In addition, the interview result supports the questionnaire result (hypothesis 9). We found that 3 interviewees (n=30, 10%) stated that concerns about the content and long-term effects of using skincare products prompted them to seek further education on short video platforms. Interviewee 1 said:

*When I first learned about skincare, I was worried because I layered a lot of products. I was also afraid that my skin would be exposed to chemicals too often and that side effects would appear. So, I looked for info on short video platforms and finally found out about skincare fasting*.

In addition, 9 interviewees (n=30, 30%) were encouraged to actively seek information through short video platforms because of their concerns over the effects of skin problems, such as acne scars, stretch marks, or irritation. As Interviewee 3 stated:

*Because I’m worried, I’m more diligent when looking for information so that I can avoid the side effects. What I’m most worried about is worsening acne. I once saw a video that said that micellar water used with cotton could make acne worse, so I tried a cleansing balm. But it turned out to be even worse*.

Furthermore, 6 interviewees (n=30, 20%) conducted an information search to minimize the side effects when the initial symptoms began to appear. As Interviewee 23 explained, “I often look for information to reduce the contrast of acne scars when I have a breakout so that acne does not leave marks.” The higher the PSE, the greater the individual’s tendency to have skin health information–seeking behavior on short video platforms. Concerns about potential long-term effects and the desire to avoid or minimize skin problems are the main factors driving users’ search for information, both as preventive and early mitigation measures before deciding to seek professional help.

### The Effect of PSU on SHEs

PSU significantly affected SHEs. This result is consistent with Malik et al [9], Zhao et al [10], and Link et al [46]. This indicates that the higher an individual’s perception of the likelihood of experiencing skin health problems, the greater the tendency to seek related information through short video platforms. Accordingly, Malik et al [9] showed that an individual’s perception of vulnerability to health conditions significantly affected preventive behavior, as individuals tend to take anticipatory steps when feeling vulnerable. Zhao et al [10] confirmed that PSU had a significant effect on health information–seeking behavior driven by the need to understand and control risk. Link et al [46] revealed that the perception of vulnerability to health problems significantly drives the search for information through online platforms.

The qualitative result also supports the findings of hypothesis 10 result. A total of 10 interviewees (n=30, 33.33%) were encouraged to be more active in seeking information as a form of preventive action because of their concerns about the potential appearance of skin problems. As Interviewee 3 stated:

*Because I’m worried, I’m more diligent when looking for information so that I can avoid the possibility of acne. I’m most afraid that I will have acne, even though my skin is fine, especially because I commute every day and am often exposed to pollution*.

In addition, because of their concerns about skin conditions, 3 interviewees (n=30, 10%) not only looked for solutions but also verified the accuracy of the information they obtained. As Interviewee 2 explained:

*Because I tend to be paranoid, I’m more diligent when looking for and verifying information so that I can get the most valid information. But yes, sometimes there’s no end because everyone has different skin. I’m most worried about having dull skin.*

Thus, the higher the individuals’ concern about the possibility of having skin problems, the greater their tendency to be actively involved in the search, verification, and application of skin health information through short video platforms.

### Theoretical Implications

This study enriched previous studies that examined the behavior of searching for health information on social media in general [9,11,15,23,24] and discussed content quality with regard to the credibility of actual information [21,22,25]. We found that the role of algorithmic ecosystems in shaping users’ skin health behavior, the shift from SC to short video platform credibility, and the importance of visual media richness in aesthetic health contexts could influence users in searching skin health information on short video platforms. The findings of this study also provide theoretical implications for SOR, MRT, and HBM theories by showing that the effectiveness of media in searching for skin health information is highly determined by media wealth, which serve as a stimulus. In the context of MRT, short videos have several media that can be used by their users, such as a combination of video, photos, audio, and caption text, making them more effective in explaining visual skin health topics. This is because the combination of media used can reduce the ambiguity and complexity of information. In the context of HBM, the visualization of skin conditions increases PSE and PSU, which encourage information-seeking behavior represent organisms and responses, respectively. This implication confirms that the effectiveness of media in health communication is not only determined by the form of the media but also by the media’s ability to trigger users’ beliefs and perceptions of skin health, as described in HBM.

This study found that HCE and PHIs serve as stimuli and had an effect on PU. This finding can expand the MRT, in which the existence of content expressiveness and personalization can make media delivery effective in the context of short videos. In addition, findings related to HCE underscore that expressiveness depends not only on the media format but also on the ways of delivery, demonstration, and visualization of skin health content. These findings are consistent with those of Priyambodo et al [11], who showed that visual appeal affects PU. This study also found that PU is also formed from a combination of content expressiveness and personalization; thus, the research model of health information–seeking behavior needs to consider visual content–based factors as the main variables. This finding is in accordance with the study by Priyambodo et al [11], which revealed that PU is a factor that drives the intention to seek health information. These findings can strengthen the relevance of the technology acceptance model and confirm the research of Malik et al [9] and Jin et al [41] regarding the relationship between the usefulness of short video platforms and information retrieval behavior.

SC in this study has no effect on SHEs so that it can expand the research of Malik et al [9] by showing that the ecosystem of short video platforms is more influenced by the relevance of content and ease of access compared to the credibility of the source. This is supported by the behavior of users who consume other content before deciding to trust the information received on a short video platform. Based on Joseph [71], most of the short video platforms governed by algorithmic processes, where this platform acts as an intermediary that could shape perceptions of identity and self-representation and amplify pressures on users to conform to idealized norms. Therefore, short video platform users need to engage in active self-construction for developing digital habits that prioritize reflective awareness, diverse media consumption, and the scrutiny of artificial intelligence recommendations to offset the passive exposure to algorithmic feedback [71,72].

The results of this study also show that through interaction features, such as likes, comments, and saves, can have a satisfaction effect on the users’ behavior of searching for skin health information. Consistent with Liu et al [4], these findings may provide further support for theories related to interactions in discussing short video platforms. In contrast to Priyambodo et al [11], who showed that user interactivity does not have a significant effect on PU, this study examined the relationship between PI and attitude.

Attitude had a weak effect on SHEs in this study. Although attitude has only a weak effect, these findings suggest that attitude’s influence on behavior is not as strong as initial assumptions and therefore requires new theoretical adaptations in the domain of short video platforms. These findings can provide insight that there are other factors that can give users a positive feeling to the users of the short video platform.

Exposure to content with certain beauty standards, such as smooth skin without acne, can trigger a process of upward comparison, which then triggers feelings of dissatisfaction with an individual’s skin condition. These findings can strengthen the research of Adkins et al [26] by confirming that USC also drives SST behavior on short video platforms. The insignificance of the effect of SST on SHEs indicates that the relationship between stigma and search behavior may be mediated by other psychological factors that have not been studied in this study. These findings may provide implications that research models related to information-seeking behavior need to integrate psychological and cognitive factors to explain the relationship between stigma and information-seeking behavior.

In the end, the strong influence of PSE and PSU on information-seeking behavior provides validation for HBM. Users who feel that their skin conditions are serious, such as inflamed acne, dark spots, and severe eczema, are likely to be encouraged to seek out skincare information, especially when they feel that the condition they are experiencing has the potential to get worse. In addition, users who have a perception of susceptibility to a disease are also encouraged to seek information to prevent getting the disease. Priyambodo et al [11] have similar results to this study; PSE and PSU have an influence on the intention to seek health information, which then affects the actual online health information–seeking behavior. These findings confirm that PSE and PSU remain the driving factors for information-seeking behavior, especially in health domains that are highly visual, such as skin health. Overall, the findings in this study show that the behavior of seeking skin health information is the result of an interaction between cognitive, psychological, social, and technological factors.

### Practical Implications

From the perspective of short video platforms, platform providers need to strengthen their role as responsible information facilitators. This can be done by providing interaction features that allow educational discussions to take place well, such as fact-check prompts. Recommendation algorithms also need to be directed to provide greater space for evidence-based educational content and corrective interaction from professionals, not just viral content. In addition, official information panels and warning labels need to be integrated into content that is vulnerable to misinterpretation so that the interaction network on the platform does not reinforce misinformation. Features that directly connect users with various trusted sources are also needed. Examples of this are displaying verified educational video recommendations, incorporating educational panels on specific dermatology keywords, and providing links to official health institutions. In this way, users can cross-check without having to leave the platform.

As content creators of health care professionals, it is important to stabilize the quality of the information circulating through their active involvement in conversations on the platform. In addition to producing educational content that is clear, visual, and easy to understand, health care workers also need to be present in digital interaction spaces, such as comment columns or video replies, to correct mistakes, answer questions, and strengthen user understanding. The presence of health care workers in the flow of public conversations on short video platforms can increase users’ confidence in accurate information. Nonprofessional creators also need to be more careful when presenting skin health information. These creators need to maintain transparency, especially in sponsored content, avoid medical claims that cannot be validated, and open up space for collaboration or clarification with health workers. By leveraging interaction features, such as duets or stitches, creators can support the spread of informative content without misleading users.

Finally, the government needs to play a role in providing official references that can make it easier for users to verify information. Governments can work with platforms to link trusted sources, such as product regulations, dangerous product lists, or clinical dermatology guidelines. In addition, health literacy programs and public education about the risks of illegal products need to be strengthened so that the public is better able to distinguish valid information from false commercial content.

### Limitations and Future Work

A limitation of this study is the demographics of the respondents, as most of the respondents were aged 20‐29 years and were taking or already had an education level equivalent to a bachelor degree. Thus, this study could not conduct a comparative study of ages. Moreover, the study did not evaluate other cognitive or psychological factors, such as prior knowledge or self-compassion, that could influence users’ behavior in searching for health information. This study did not conduct an analysis based on the type of skin health information—that is, primary information on medical and tertiary conditions and aesthetics oriented toward beauty care.

Further studies could explore the effects of SST and SC on more general research objects, such as general health, on short video platforms. Specific content differentiation is also necessary, given that skin health information has diverse types of information, such as product reviews, skin health education, and information related to skin diseases, which are likely to drive different behaviors.

## Supplementary material

10.2196/93461 Multimedia Appendix 1 Measurement items in the questionnaire.

10.2196/93461 Multimedia Appendix 2 Questionnaire respondents’ demographics.

10.2196/93461 Checklist 1 CHERRIES checklist.

## Abbreviations

AVE: average extracted variance
CA: Cronbach α
CFA: confirmatory factor analysis
CHERRIES: Checklist for Reporting Results of Internet E-Surveys
CMB: common method bias
GoF: goodness of fit
HBM: health belief model
HCE: health content expressiveness
MRT: media richness theory
PHI: personalized health insight
PI: perceived interactivity
PSE: perceived severity
PSU: perceived susceptibility
PU: perceived usefulness
SC: source credibility
SHE: skin health information seeking on short video platform
SOR: stimulus-organism-response
SST: skin stigmatization
USC: upward skin comparison

## References

[1] Schumacher S, Sparks G, Montalvo I, Kirzinger A, Hamel L (2025). KFFtracking poll on health information and trust: health information and advice on social media. KFF.

[2] Vranica S, Kruppa M (2024). Google’s grip on search slips as TikTok and AI startup mount challenge. WSJ; The Wall Street Journal.

[3] (2025). TikTok what’s next 2025 trend report: it’s not magic, it’s chemistry—introducing “Brand Chem”. TikTok.

[4] Liu J, Huang R, Ren J, Li P, Wang P (2025). The intention to use short videos for health information among Chinese adults: based on the technology acceptance model. Digit Health.

[5] Ploderer B, Rezaei Aghdam A, Burns K (2022). Patient-generated health photos and videos across health and well-being contexts: scoping review. J Med Internet Res.

[6] Daft RL, Lengel RH (1986). Organizational information requirements, media richness and structural design. Manage Sci.

[7] Han P, Liu S, Zhang D, Li X, Li X (2025). Research on the factors affecting the adoption of health short videos by the college students in China: unification based on TAM and UTAUT model. Front Psychol.

[8] Rosenstock IM, Strecher VJ, Becker MH (1988). Social learning theory and the Health Belief Model. Health Educ Q.

[9] Malik A, Islam T, Ahmad M, Mahmood K (2023). Health information seeking and sharing behavior of young adults on social media in Pakistan. J Librariansh Inf Sci.

[10] Zhao YC, Zhao M, Song S (2022). Online health information seeking among patients with chronic conditions: integrating the Health Belief Model and Social Support Theory. J Med Internet Res.

[11] Priyambodo AZ, Zhafirah NA, Shabur R, Handayani PW, Fitriani H (2025). Health information-seeking using short video platforms. Digit Health.

[12] Hansen S, Jensen TS, Schmidt AM, Strøm J, Vistisen P, Høybye MT (2024). The effectiveness of video animations as a tool to improve health information recall for patients: systematic review. J Med Internet Res.

[13] Zhu Z, Liu S, Zhang R (2023). Examining the persuasive effects of health communication in short videos: systematic review. J Med Internet Res.

[14] (2025). Survei penetrasi internet dan perilaku penggunaan internet 2025. APJII.

[15] Thang CJ, Garate D, Thang J, Lipoff JB, Barbieri JS (2023). Short-form medical media: a multi-platform analysis of acne treatment information in TikTok videos, Instagram reels, and YouTube shorts. JMIR Dermatol.

[16] Zhang J, Yuan J, Zhang D (2024). Short video platforms as sources of health information about cervical cancer: a content and quality analysis. PLoS One.

[17] Ming S, Han J, Yao X, Guo X, Guo Q, Lei B (2024). Myopia information on TikTok: analysis factors that impact video quality and audience engagement. BMC Public Health.

[18] Kirkpatrick CE, Lawrie LL (2024). TikTok as a source of health information and misinformation for young women in the United States: survey study. JMIR Infodemiology.

[19] Nagara MRND, Nurhajati L (2022). The construction and adoption of beauty standard by youth female as the consumer of K-beauty products in Indonesia. Jurnal Riset Komunikasi.

[20] Ariana H, Almuhtadi I, Natania NJ, Handayani PW, Bressan S, Larasati PD (2024). Influence of TikTok on body satisfaction among generation Z in Indonesia: mixed methods approach. JMIR Hum Factors.

[21] Khan S, Yee D, Khan S (2022). Biologics to breast milk: a cross-sectional study of popular eczema treatment content on TikTok. Pediatr Dermatol.

[22] Awad N, Hetzel J, Bhupalam V, Nestor MS (2024). A cross‐sectional content quality analysis of information in TikTok videos on “Dermarolling (Roller Microneedling)”. J Cosmet Dermatol.

[23] Yu M, Guo Y, Wang J, Liu X (2025). Antecedents of health short videos’ information adoption and continuous usage intention: evidence from SEM and fsQCA. IMDS.

[24] Zhang X, Chen B, Li G, Dong Y (2022). Exploring the health information seeking behavior of social media users under the background of COVID-19 pandemic: an empirical study based on social cognitive theory. Front Psychol.

[25] Subramanyam C, Becker A, Rizzo J, Afzal N, Nong Y, Sivamani R (2024). Visibility of board-certified dermatologists on TikTok. JMIR Dermatol.

[26] Adkins K, Overton PG, Moses J, Thompson A (2023). Investigating the role of upward comparisons and self-compassion on stigma in people with acne: cross-sectional study. JMIR Dermatol.

[27] Brzozowska JM, Gotlib J (2025). Social media potential and impact on changing behaviors and actions in skin health promotion: systematic review. J Med Internet Res.

[28] Hu B, Zhu Y, Bao R, Zhao Z, Liu C, Lin A (2025). Social media, health consciousness, and cultural influences on sugar reduction behaviors in Chinese youth: extending the stimulus-organism-response model. J Med Internet Res.

[29] Ahmad K, Lilani K (2025). From scrolling to buying: role of beauty influencers on impulse buying a stimulus-organism-response perspective. Telemat Inform Rep.

[30] Niu Z, Willoughby JF, Coups EJ, Stapleton JL (2021). Effects of website interactivity on skin cancer-related intentions and user experience: factorial randomized experiment. J Med Internet Res.

[31] Tseng FC, Huang TL, Pham TTL, Cheng TCE, Teng CI (2022). How does media richness foster online gamer loyalty?. Int J Inf Manage.

[32] Lin SH, Lin TMY (2018). Demand for online platforms for medical word-of-mouth. J Int Med Res.

[33] Lenczowski E, Tung-Hahn E, Higareda J (2018). Video education to improve recognition of common benign and malignant cutaneous lesions and skin cancer prevention in the public. Int J Womens Dermatol.

[34] Zhang X, Guo X, Guo F, Lai KH (2014). Nonlinearities in personalization-privacy paradox in mHealth adoption: the mediating role of perceived usefulness and attitude. Technol Health Care.

[35] Nittas V, Mütsch M, Frey T, Braun J, Puhan MA (2022). Effectiveness of a tailored web app on sun protection intentions and its implications for skin cancer prevention: a randomized controlled trial. PLoS Digit Health.

[36] Yang H, Zhang S, Diao Z, Sun D (2023). What motivates users to continue using current short video applications? A dual-path examination of flow experience and cognitive lock-in. Telemat Inform.

[37] Haykal D (2025). Digital twins in dermatology: a new era of personalized skin care. Front Digit Health.

[38] Xia L, Deng S, Liu Y (2017). Seeking health information online: the moderating effects of problematic situations on user intention. J Data Inform Sci.

[39] Boyce L, Harun A, Prybutok G, Prybutok VR (2024). The role of technology in online health communities: a study of information-seeking behavior. Healthcare (Basel).

[40] Kim J, Park HA (2012). Development of a health information technology acceptance model using consumers’ health behavior intention. J Med Internet Res.

[41] Jin XL, Yin M, Zhou Z, Yu X (2021). The differential effects of trusting beliefs on social media users’ willingness to adopt and share health knowledge. Inf Process Manag.

[42] Claggett JL, Kitchens B, Paino M (2024). Identifying the peripheral cues in the credibility assessment of online health information. Inf Manag.

[43] Fan F, Chan K, Tsang L (2024). Health information-seeking behavior and perceived information source credibility among middle-aged and older adults. Health New Media Res.

[44] McMillan SJ, Hwang JS (2002). Measures of perceived interactivity: an exploration of the role of direction of communication, user control, and time in shaping perceptions of interactivity. J Advert.

[45] Liu C, Jiang M, Muhammad ZA (2024). The impact of TikTok short video factors on tourists’ behavioral intention among generation Z and millennials: the role of flow experience. PLoS One.

[46] Link E, Baumann E, Klimmt C (2021). Explaining online information seeking behaviors in people with different health statuses: German representative cross-sectional survey. J Med Internet Res.

[47] Mirzaei A, Aslani P, Luca EJ, Schneider CR (2021). Correction: predictors of health information–seeking behavior: systematic literature review and network analysis. J Med Internet Res.

[48] Li H, Li D, Zhai M, Lin L, Cao Z (2025). Associations among online health information seeking behavior, online health information perception, and health service utilization: cross-sectional study. J Med Internet Res.

[49] Neely S, Eldredge C, Sanders R (2021). Health information seeking behaviors on social media during the COVID-19 pandemic among American social networking site users: survey study. J Med Internet Res.

[50] Tian J, Li B, Zhang R (2024). The impact of upward social comparison on social media on appearance anxiety: a moderated mediation model. Behav Sci (Basel).

[51] Van Beugen S, Schut C, Kupfer J (2023). Perceived stigmatization among dermatological outpatients compared with controls: an observational multicentre study in 17 European countries. Acta Derm Venereol.

[52] Tunca M, Balik ZB, Uncu HB, Botsali A (2025). Stigmatization and psychosocial burden in acne patients. J Cosmet Dermatol.

[53] Berger M, Wagner TH, Baker LC (2005). Internet use and stigmatized illness. Soc Sci Med.

[54] Aslan Kayıran M, Karadağ AS, Oğuz Topal İ (2022). Habits of using social media and the internet in psoriasis patients. Dermatol Pract Concept.

[55] Nguyen SH, Vu GT, Nguyen LH (2020). Understanding social media use and engagement among dermatology patients to inform dermatological prevention and care in Vietnam: cross-sectional study. JMIR Dermatol.

[56] Zhao Y, Zhang J (2017). Consumer health information seeking in social media: a literature review. Health Info Libr J.

[57] Ginsburg IH, Link BG (1989). Feelings of stigmatization in patients with psoriasis. J Am Acad Dermatol.

[58] Stangl AL, Earnshaw VA, Logie CH (2019). The health stigma and discrimination framework: a global, crosscutting framework to inform research, intervention development, and policy on health-related stigmas. BMC Med.

[59] Rowlands IJ, Loxton D, Dobson A, Mishra GD (2015). Seeking health information online: association with young Australian women’s physical, mental, and reproductive health. J Med Internet Res.

[60] Silver RA, Johnson C (2023). Health information seeking behavior on social networking sites and self-treatment: pilot survey study. Online J Public Health Inform.

[61] Griffin RJ, Dunwoody S, Yang ZJ (2013). Linking risk messages to information seeking and processing. Ann Int Commun Assoc.

[62] Richard MA, Saint Aroman M, Baissac C (2023). Burden of visible [face and hands] skin diseases: results from a large international survey. Ann Dermatol Venereol.

[63] Zhou X, Roberto AJ (2022). An application of the risk information seeking and processing model in understanding college students’ COVID-19 vaccination information seeking and behavior. Science Communication.

[64] Nowell LS, Norris JM, White DE, Moules NJ (2017). Thematic analysis: striving to meet the trustworthiness criteria. Int J Qual Methods.

[65] Podsakoff PM, MacKenzie SB, Lee JY, Podsakoff NP (2003). Common method biases in behavioral research: a critical review of the literature and recommended remedies. J Appl Psychol.

[66] Fuller CM, Simmering MJ, Atinc G, Atinc Y, Babin BJ (2016). Common methods variance detection in business research. J Bus Res.

[67] Hair JF, Babin BJ, Ringle CM, Sarstedt M, Becker JM (2025). Covariance-based structural equation modeling (CB-SEM): a SmartPLS 4 software tutorial. J Market Anal.

[68] Armstrong AW, Kim RH, Idriss NZ, Larsen LN, Lio PA (2011). Online video improves clinical outcomes in adults with atopic dermatitis: a randomized controlled trial. J Am Acad Dermatol.

[69] Ma T, Atkin D (2017). User generated content and credibility evaluation of online health information: a meta analytic study. Telemat Inform.

[70] Denniss E, Lindberg R, McNaughton SA (2022). Development of principles for health-related information on smedia: Delphi study. J Med Internet Res.

[71] Joseph J (2025). The algorithmic self: how AI is reshaping human identity, introspection, and agency. Front Psychol.

[72] Shin D (2026). Automating epistemology: how AI reconfigures truth, authority, and verification. AI Soc.

[73] Hossain MS, Islam T, Babu M (2025). The influence of celebrity credibility, attractiveness, and social media influence on trustworthiness, perceived quality, and purchase intention for natural beauty care products. Clean Responsible Consum.

[74] Huang W, Wang X, Zhang Q, Han J, Zhang R (2025). Beyond likes and comments: how social proof influences consumer impulse buying on short-form video platforms. J Retail Consum Serv.

[75] Hussain B, Aslam S, Imran A (2025). Manufacturing beauty: how AI and social media are redefining aesthetic norms in emerging digital cultures. Acta Psychol (Amst).

