# Supplementary material for: Skin Health Information Seeking on Short Video Platforms in Indonesia: Mixed Methods Approach
Source: JMIR Dermatol. 2026 Jul 3;9:e93461. doi: 10.2196/93461 (PMC13331247; doi:10.2196/93461)
Supplement: Multimedia Appendix 1 [file derma-v9-e93461-s001.docx]

## **Table S1. Measurement items in the questionnaire.**

| **Code** | **Measurement Items** | **References** |
| --- | --- | --- |
| HCE1 | I can receive and understand skin health information on the short video platform through the verbal expression of the content creator. | [1] |
| HCE2 | I can receive and understand skin health information on the short video platform through written context. |  |
| HCE3 | I was able to receive and understand skin health information on the short video platform through the body language and facial expressions of the content creator. |  |
| HCE4 | I can receive and understand skin health information on short video platforms through images, video clips, emojis, and other symbols. |  |
| PHI1 | I can choose skin health information videos on the short video platform with different forms of presentations, symbols, and themes as per my needs. |  |
| PHI2 | I can give advice to skin health information content creators on short video platforms to publish the information I need through private messages and comments. |  |
| PHI3 | I judged the skin health information videos on the short video platform recommended to me to reflect my needs. |  |
| PHI4 | I feel the skin health information videos on the short video platform recommended to me suit my needs. |  |
| PU1 | I feel that skin health information videos on short video platforms can improve my ability to maintain healthy skin. |  |
| PU2 | I feel that skin health information videos on short video platforms can improve my ability to better address skin health issues in real life. |  |
| PU3 | I feel that skin health information videos on short video platforms can improve my efficiency in getting skin health information. |  |
| PU4 | I feel that skin health information videos on short video platforms can increase my effectiveness in getting skin health information. | [2] |
| PU5 | I feel that skin health information videos on short video platforms can help keep my skin healthy. |  |
| SC1 | I assess that the creators of skin health information content that I see on short video platforms have knowledge of the skin health topics being discussed. |  |
| SC2 | I consider the creators of skin health information content that I see on the short video platform to be experts on the topic of skin health being discussed. |  |
| SC3 | I consider the creators of skin health information content that I see on short video platforms to be trustworthy in providing skin health information. |  |
| SC4 | I rate the creators of skin health information content I see on short video platforms to be reliable in providing skin health information. |  |
| SC5 | I assess the creators of skin health information content that I see on short video platforms to have a good reputation for providing skin health information. |  |
| PI1 | I feel like I can set up the kind of skin health information videos I want to see on the short video platform. |  |
| PI2 | I feel like users of the short video platform share their experiences with each other about skin health information videos. |  |
| PI3 | I felt that the skin health information videos displayed were in line with my expectations, when I interacted (liked/commented/watched) with skin health information videos on the short video platform. |  |
| PI4 | I feel that the skin health information videos that are displayed are more relevant to my needs when I interact (like/comment/watch) with skin health information videos on short video platforms. |  |
| AT1 | I think that using a short video platform to search for skin health information is a good decision. |  |
| AT2 | I feel good when I use short video platforms to search for skin health information. |  |
| AT3 | I think that using a short video platform to search for skin health information is a wise decision. |  |
| AT4 | I rate the skin health information content on the short video platform as a valuable/useful tool for me. |  |
| USC1 | I tend to compare my skin condition to people who I think have better skin conditions. |  |
| USC2 | I tend to wonder how I can 'match' someone with a healthy skin condition (e.g. acne-free, irritation-free, or large spots), when I see them. |  |
| USC3 | I tend to wonder if my skin condition is healthy (e.g. acne-free, irritation-free, or large spots), like the skin condition I see on short video platforms. |  |
| USC4 | I found myself thinking about whether my own skin condition was comparable to the skin condition of models and celebrities. |  |
| SST1 | I find myself less attractive than most others because of my skin health issues. |  |
| SST2 | I feel anxious because of what others might think about my skin health issues. |  |
| SST3 | I hope to improve my skin health problems drastically. |  |
| SST4 | I feel like my skin health issues are the main way I judge myself. |  |
| SST5 | I don't feel like my true self because of my skin health issues. |  |
| PSE1 | I take the consequences of the skin health problems discussed on the short video platform seriously. | [3] |
| PSE2 | I assume that the skin health issues discussed on the short video platform can cause me a big problem, if I experience them. |  |
| PSE3 | I am afraid of having skin health problems as discussed on the short video platform. |  |
| PSE4 | I consider the effort to avoid the skin health problems discussed on the short video platform important. |  |
| PSU1 | I have a high chance of experiencing skin health problems discussed on short video platforms. |  |
| PSU2 | I was worried about the possibility of me having skin health problems as discussed on the short video platform. |  |
| PSU3 | I feel that I have the possibility of experiencing skin health problems as discussed in the short video platform. |  |
| PSU4 | I have a family that has issues related to skin health as discussed on the short video platform. | [4] |
| PSU5 | I have a high probability of having skin health problems as discussed on the short video platform due to the bad habits I have been doing. |  |
| SHE1 | I use the short video platform to search for information and descriptions related to skin health topics. |  |
| SHE2 | I intend to continue to seek out skin health information through short video platforms when needed. | [3] |
| SHE3 | I am willing to constantly search for skin health information on short video platforms on topics that interest me. |  |
| SHE4 | I intend to keep looking for skin health information on short video platforms in the future. |  |
| SHE5 | I am willing to consider the skin health experiences of other users on the short video platform before making a treatment decision. |  |

**References**

[1] Yu M, Guo Y, Wang J, Liu X. Antecedents of health short videos’ information adoption and continuous usage intention: evidence from SEM and fsQCA. *Industrial Management & Data Systems* 2025. <https://doi.org/10.1108/imds-06-2024-0569>

[2] Liu J, Huang R, Ren J, Li P, Wang P. The intention to use short videos for health information among Chinese adults: Based on the technology acceptance model. Digit Health. 2025 Apr 15;11:20552076251335519. doi: 10.1177/20552076251335519

[3] Malik A, Islam T, Ahmad M, Mahmood K. Health information seeking and sharing behavior of young adults on social media in Pakistan. *Journal of Librarianship and Information Science*. 2022;*55*(3):096100062210902. <https://doi.org/10.1177/09610006221090228>

[4] Zhang X, Guo X, Guo F, Lai KH. Nonlinearities in personalization-privacy paradox in mHealth adoption: the mediating role of perceived usefulness and attitude. Technol Health Care. 2014;22(4):515-29. doi: 10.3233/THC-140811
