# Supplementary material for: Skin Health Information Seeking on Short Video Platforms in Indonesia: Mixed Methods Approach
Source: JMIR Dermatol. 2026 Jul 3;9:e93461. doi: 10.2196/93461 (PMC13331247; doi:10.2196/93461)
Supplement: Multimedia Appendix 2 [file derma-v9-e93461-s002.docx]

**Table S1. Questionnaire respondents’ demographics.**

| **Demographic Variables** | | **Number of Respondents** | **Percentage** |
| --- | --- | --- | --- |
| Gender | Male | 251 | 41.63% |
|  | Women | 352 | 58.37% |
| Age | 20-24 years old | 329 | 54.56% |
|  | 25-29 years old | 105 | 17.41% |
|  | 30-34 years old | 63 | 10.45% |
|  | 35-39 years old | 36 | 5.97% |
|  | 40-44 years old | 22 | 3.65% |
|  | 45-49 years old | 15 | 2.49% |
|  | ≥ 50 years old | 33 | 5.47% |
| Recent or current level of education | High school | 73 | 12.11% |
|  | Diploma | 32 | 5.31% |
|  | Bachelor | 466 | 77.28% |
|  | Master | 32 | 5.31% |
| Jobs | Student | 297 | 49.25% |
|  | Housewives | 43 | 7.13% |
|  | Civil servants | 25 | 4.15% |
|  | Private employees | 171 | 28.36% |
|  | Stated-owned employees | 18 | 2.99% |
|  | Self-employed | 26 | 4.31% |
|  | Health workers | 4 | 0.66% |
|  | Unemployment | 5 | 0.83% |
|  | Not mentioned | 5 | 0.83% |
|  | Educators | 4 | 0.66% |
|  | Others | 5 | 0.83% |
| Domicile | Greater Jakarta | 382 | 63.35% |
|  | Java Island other than Greater Jakarta | 164 | 27.20% |
|  | Sumatra, Bangka Belitung, and Riau Islands | 38 | 6.30% |
|  | Sulawesi | 7 | 1.16% |
|  | Kalimantan | 9 | 1.49% |
|  | Bali and Nusa Tenggara | 3 | 0.50% |
